# 3D T2-weighted imaging to shorten multiparametric prostate MRI protocols

**DOI:** 10.1007/s00330-017-5120-5

**Published:** 2017-11-13

**Authors:** Stephan H. Polanec, Mathias Lazar, Georg J. Wengert, Hubert Bickel, Claudio Spick, Martin Susani, Shahrokh Shariat, Paola Clauser, Pascal A. T. Baltzer

**Affiliations:** 1 Department of Biomedical Imaging and Image-Guided Therapy, Medical University of Vienna, A-1090, Wien, Vienna, Austria; 2 Clinical Institute of Pathology, Medical University of Vienna, Vienna, Austria; 3 Department of Urology, Medical University of Vienna (AKH), Waehringer-Guertel 18-20, A-1090 Vienna, Austria; 4 Christian Doppler Laboratory for Medical Radiation Research for Radiation Oncology, Medical University of Vienna, Vienna, Austria

**Keywords:** Prostate, MRI, Abbreviated protocol, 3D imaging, Multiparametric MRI

## Abstract

**Objectives:**

To determine whether 3D acquisitions provide equivalent image quality, lesion delineation quality and PI-RADS v2 performance compared to 2D acquisitions in T2-weighted imaging of the prostate at 3 T.

**Methods:**

This IRB-approved, prospective study included 150 consecutive patients (mean age 63.7 years, 35–84 years; mean PSA 7.2 ng/ml, 0.4–31.1 ng/ml). Two uroradiologists (R1, R2) independently rated image quality and lesion delineation quality using a five-point ordinal scale and assigned a PI-RADS score for 2D and 3D T2-weighted image data sets. Data were compared using visual grading characteristics (VGC) and receiver operating characteristics (ROC)/area under the curve (AUC) analysis.

**Results:**

Image quality was similarly good to excellent for 2D T2w (mean score R1, 4.3 ± 0.81; R2, 4.7 ± 0.83) and 3D T2w (mean score R1, 4.3 ± 0.82; R2, 4.7 ± 0.69), *p* = 0.269. Lesion delineation was rated good to excellent for 2D (mean score R1, 4.16 ± 0.81; R2, 4.19 ± 0.92) and 3D T2w (R1, 4.19 ± 0.94; R2, 4.27 ± 0.94) without significant differences (*p* = 0.785). ROC analysis showed an equivalent performance for 2D (AUC 0.580–0.623) and 3D (AUC 0.576–0.629) T2w (*p* > 0.05, respectively).

**Conclusions:**

Three-dimensional acquisitions demonstrated equivalent image and lesion delineation quality, and PI-RADS v2 performance, compared to 2D in T2-weighted imaging of the prostate. Three-dimensional T2-weighted imaging could be used to considerably shorten prostate MRI protocols in clinical practice.

***Key points*:**

*• 3D shows equivalent image quality and lesion delineation compared to 2D T2w.*

*• 3D T2w and 2D T2w image acquisition demonstrated comparable diagnostic performance.*

*• Using a single 3D T2w acquisition may shorten the protocol by 40%.*

*• Combined with short DCE, multiparametric protocols of 10 min are feasible.*

## Introduction

Multiparametric MRI (MP-MRI) of the prostate is increasingly recognised as an accurate tool for the diagnosis of prostate cancer (PCa) [1]. It enables both detection and risk stratification with regard to the biological aggressiveness of prostate cancer in different clinical settings [2–5]. This growing acceptance of MP-MRI has substantially increased the frequency of MP-MRI examinations of the prostate [6]. To provide an MP-MRI imaging service to all men who might profit from it, a standardised and as-short-as-possible MP-MRI protocol is required.

Currently, combinations of high-resolution, T2-weighted imaging, diffusion-weighted imaging (DWI) and dynamic contrast-enhanced (DCE) imaging are key components for accurate interpretation and reporting of prostate findings [7, 8]. While MP-MRI of prostate is considered an accurate and cost-effective tool for the detection of PCa [9], it is also an expensive technology. In times of increasing healthcare costs and decreasing healthcare budgets, a certain need to optimise the number of patients who can be scanned per unit of time is evident. One elegant way to considerably shorten the imaging protocol without losing a diagnostic parameter would be to exchange several multiplanar two-dimensional acquisitions of T2-weighted images by a single, isotropic 3D acquisition that can be used for subsequent multiplanar reconstructions. Currently, such acquisitions are considered solely as an adjunct to 2D acquisitions [8].

Consequently, the purpose of our study was to determine whether 3D T2-weighted image acquisitions achieve similar image quality, lesion delineation quality and PI-RADS v2 performance compared to standard 2D acquisitions in prostate MRI at 3 T, without an endorectal coil.

## Materials and methods

### Patients

The local ethics committee approved this prospective study. Between November 2015 and February 2017, 150 consecutive patients fulfilled the inclusion criteria: multiparametric MRI of the prostate due to elevated PSA level and/or a suspicious digital rectal examination and/or local staging of the cancer in case of known prostate cancer. There were no specific exclusion criteria.

### MR imaging

All patients were examined using a 3-T MRI system (Tim Trio, Siemens Healthineers, Erlangen, Germany). The vendor-supplied combined spine array and body array receive-only coils were used for signal acquisition. After emptying the bladder, the patients were positioned in the feet-first supine position. An antiperistaltic agent, 10 mg of hyoscine butyl-bromide (Buscopan®, Boehringer Ingelheim, GmbH, Germany), was injected intramuscularly. In addition, the rectum was filled with ultrasound gel to improve image quality by minimising artefacts.

The imaging protocol was performed in accordance with the PI-RADS v2 guidelines, including T2w images, diffusion-weighted imaging (DWI) and dynamic contrast enhancement (DCE) [8]. Detailed image acquisition parameters are displayed in Table 1.

**Table 1:** Parameters for 3-T images

Sequence	Spatial resolution (mm)	TR (ms)	TE (ms)	FOV (mm)	Averages	Flip angle	TA (min:s)
2D T2 TSE axial	0.6 × 0.6 × 3	4000	101	200	3	150	4:10
2D T2 TSE sagittal	0.6 × 0.6 × 3	4000	101	200	3	150	3:46
2D T2 TSE Coronal	0.6 × 0.6 × 3	4000	101	200	3	150	3:38
					Total time		11:14
3D T2 SPACE	0.8 × 0.8 × 0.8	1800	179	320	2	100	3:45
					Total time		3:35
DWI	1.6 × 1.6 × 3.6	3300	60	260	8		2:29
T1 TWIST DCE	1 × 1 × 3.6	3.85	1.42	260	1	2.5/10/20	5:02
					Total time		7:31

2D two-dimensional, DWI diffusion weighted imaging, FOV field of view, SPACE Sampling Perfection with Application optimized Contrasts using different flip angle Evolution, TA time of acquisition, TE echo time, TR repetition time, TSE turbo spin echo, TWIST Time-resolved angiography With Interleaved Stochastic Trajectories; all sequences were acquired with GeneRalized Autocalibrating Partially Parallel Acquisition (GRAPPA) factor 2

The image acquisition time for the three-parametric protocol was approximately 19 min comprising 11:14 min for 2D T2w, 2:29 min for DWI and 5:02 min for DCE.

#### Image analysis

Qualitative visual image analysis was performed by two different radiologists with at least 5 years of experience in MRI (S.P., M.L.). The image analysis was performed on a dedicated workstation. The images were anonymised and reviewed randomly. To avoid any bias, the two readers independently reviewed each acquired data set and assigned ordinal numeric scores to each examination.

Lesions that were noted in the report were independently assessed by each reviewer. Lesion delineation was rated from 1 (poor) to 5 (excellent) and a diagnostic PI-RADS category was assigned for the most suspicious lesion.

##### Overall image quality

A score ranging from 1 to 5 was used to describe the overall image quality:

- 1 = images non-diagnostic
- 2 = images poor but still interpretable
- 3 = image quality acceptable
- 4 = image quality good
- 5 = image quality excellent

**Fig. 1:**
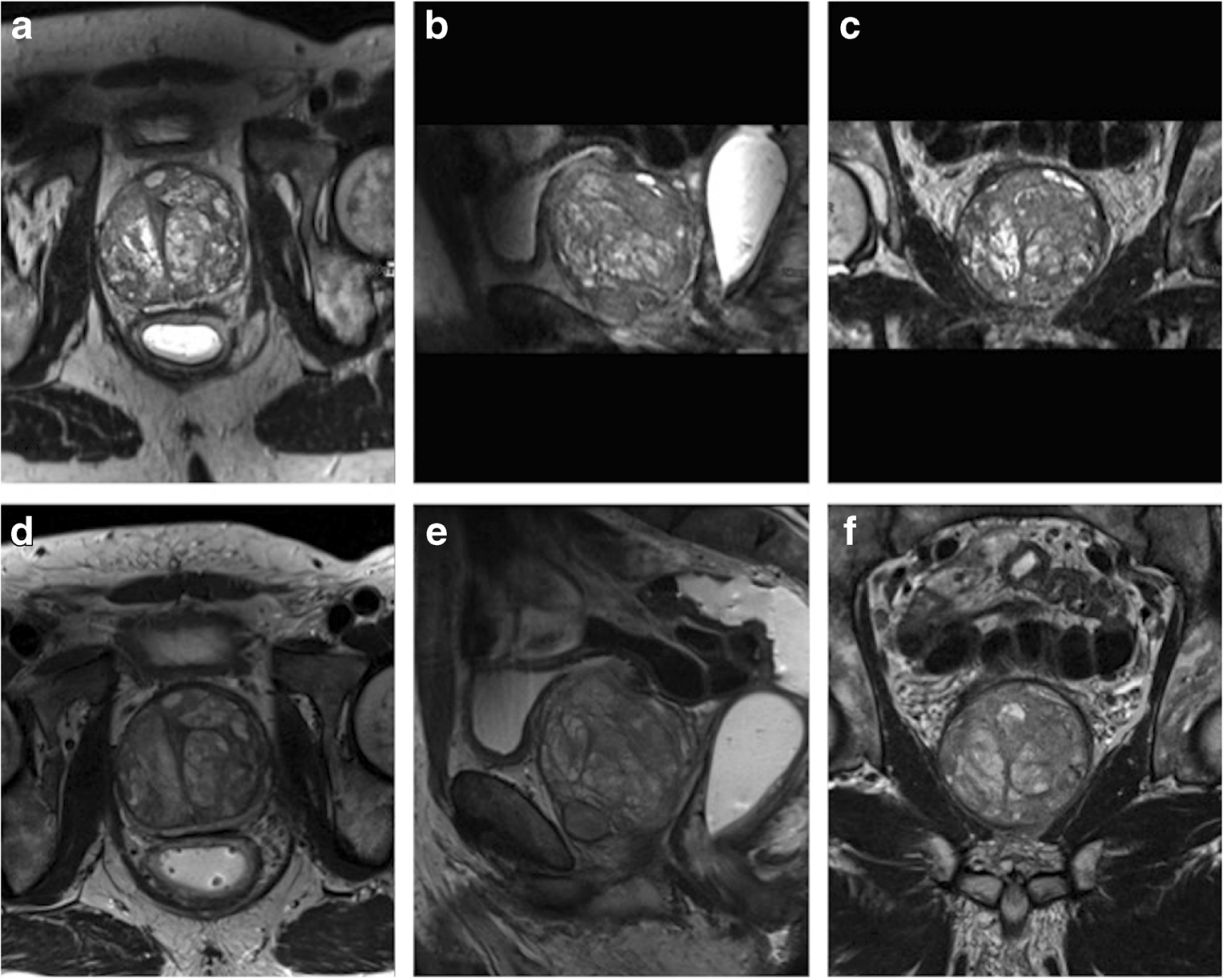
Examples of image quality grading. Images were obtained in a 67-year-old man (PSA 7.38 ng/ml) with benign prostate hyperplasia. a–c 3D T2w images displayed in axial, coronal and sagittal plane. d–f 2D acquired T2w images in coronal, sagittal and axial plane. Both readers scored the image quality for both techniques excellent (score 5 on the Likert scale). No suspicious lesion was detected in this patient

An example of image quality grading is provided in Fig. 1.

##### Lesion delineation

The readers evaluated the delineation of the lesions and assigned a confidence score ranging from 1 to 5. In patients with more than one lesion, we assessed an index lesion. The index lesion was determined by the largest and best-seen lesion.

- 1 = non-diagnostic
- 2 = poor but still interpretable
- 3= acceptable
- 4 = good
- 5 = excellent

**Fig. 2:**
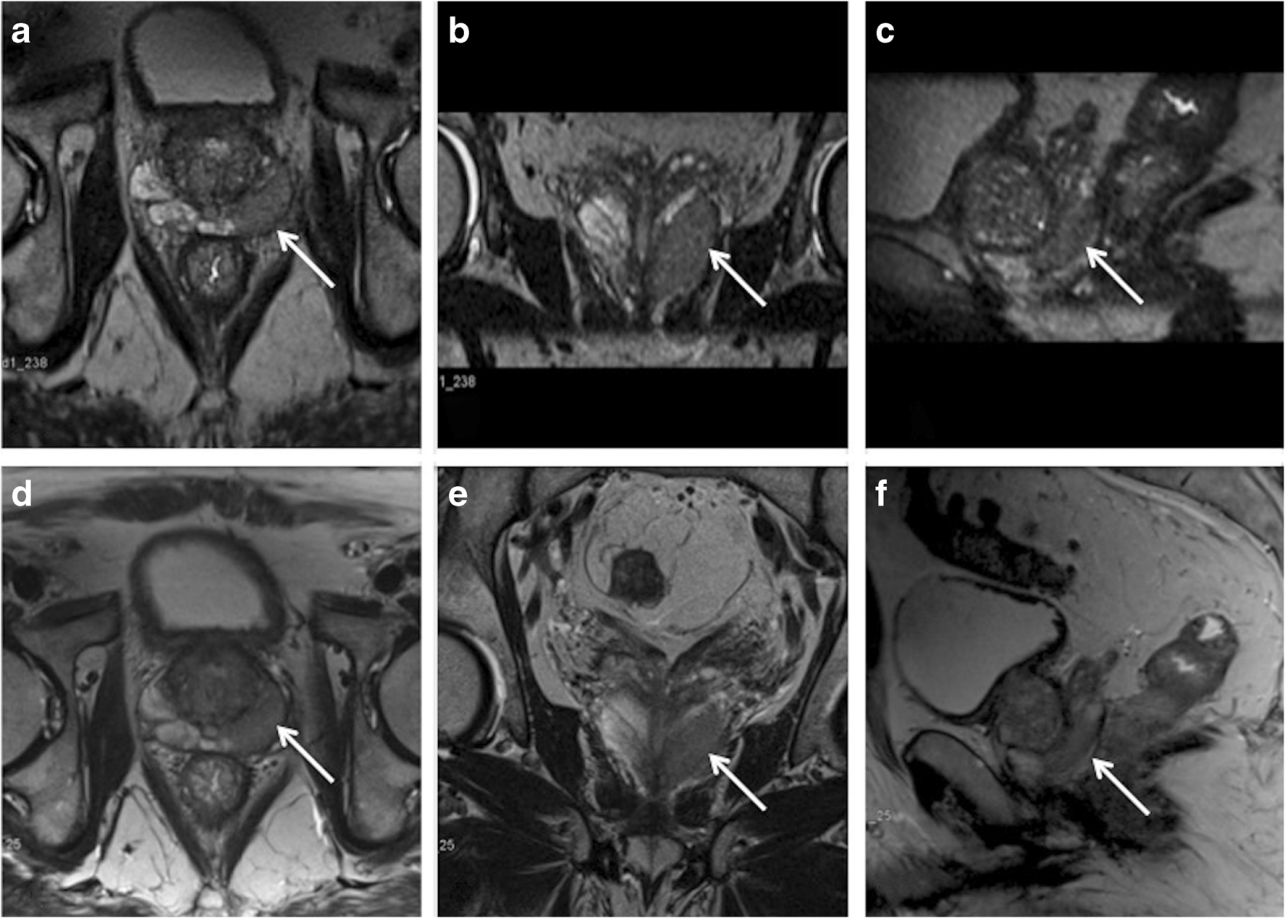
Examples of lesion delineation and characterisation. Images were obtained in a 73-year-old (PSA 9.28 ng/ml) biopsy-naïve man. a–c 3D T2w images displayed in coronal, sagittal and axial plane. d–f 2D acquired T2w images in axial, coronal and sagittal plane. Both readers scored the image quality for both techniques excellent (score 5 on the Likert scale). A lesion was detected in the left peripheral zone (arrow). For lesion delineation the score was excellent and both readers rated the lesion as PI-RADS 5. Histopathology obtained by MRI-guided biopsy confirmed a PCa Gleason score 8 (4 + 4)

An example of lesion delineation and characterisation is provided in Fig. 2.

##### Lesion characterisation

For lesion characterisation, the assessed lesions were rated according to the PI-RADS version 2 guidelines [8]. Both readers independently scored the lesions on 2D and 3D T2 acquisition images in a separate reading session (Fig. 2).

#### Standard of reference for lesion diagnosis

For lesion diagnosis, only histologically verified cases were considered. Histopathology was obtained as follows: in case a lesion was assigned a PI-RADS 4/5 category, MRI-guided, in-bore 18G core biopsy was performed according to reported standards [10]. If lesions were biopsied before prostate MRI, lesion location was correlated between MRI images and histopathology. In case such alignment was impossible, the lesion was considered as not having a diagnostic standard of reference.

### Statistical analysis

All the statistical analyses were performed with commercially available software. For data collection and management, an Excel 2011 (Microsoft Corporation, Washington, USA) spreadsheet was used. The statistical analysis was performed with Medcalc 15.8 (Medcalc Software bvba, Ostend, Belgium). Values of *p* < 0.05 were considered statistically significant.

Ordinal visual analysis ratings were analysed by visual grading characteristics (VGC) analysis, a non-parametric, rank-invariant statistical method derived from receiver operating characteristics (ROC) analysis. VGC was used to directly determine the difference in image quality between the two T2w image acquisitions [11]. The area under the VGC curve (AUC) was expected to be a single measure of the difference in image quality when comparing two or more modalities [11]. The range value for the AUC is 0.5–1.0, where 0.5 represents no superiority of a method with regard to the rated image quality criterion, while 1.0 represents absolute superiority of the respective method with regard to the examined image quality criterion [11]. ROC analysis was performed to compare the diagnostic performance of PI-RADS scores between 2D and 3D T2w acquisitions. Values of *p* < 0.05 were considered statistically significant.

## Results

Between November 2015 and February 2017, 150 consecutive patients (mean age 63.7 years, 35–84 years; mean PSA 7.2 ng/ml, 0.4–31.1 ng/ml) met the inclusion criteria. No patients had to be excluded. In all consecutive patients, the examination was conducted without interruptions or side effects.

### Overall image quality

**Fig. 3:**
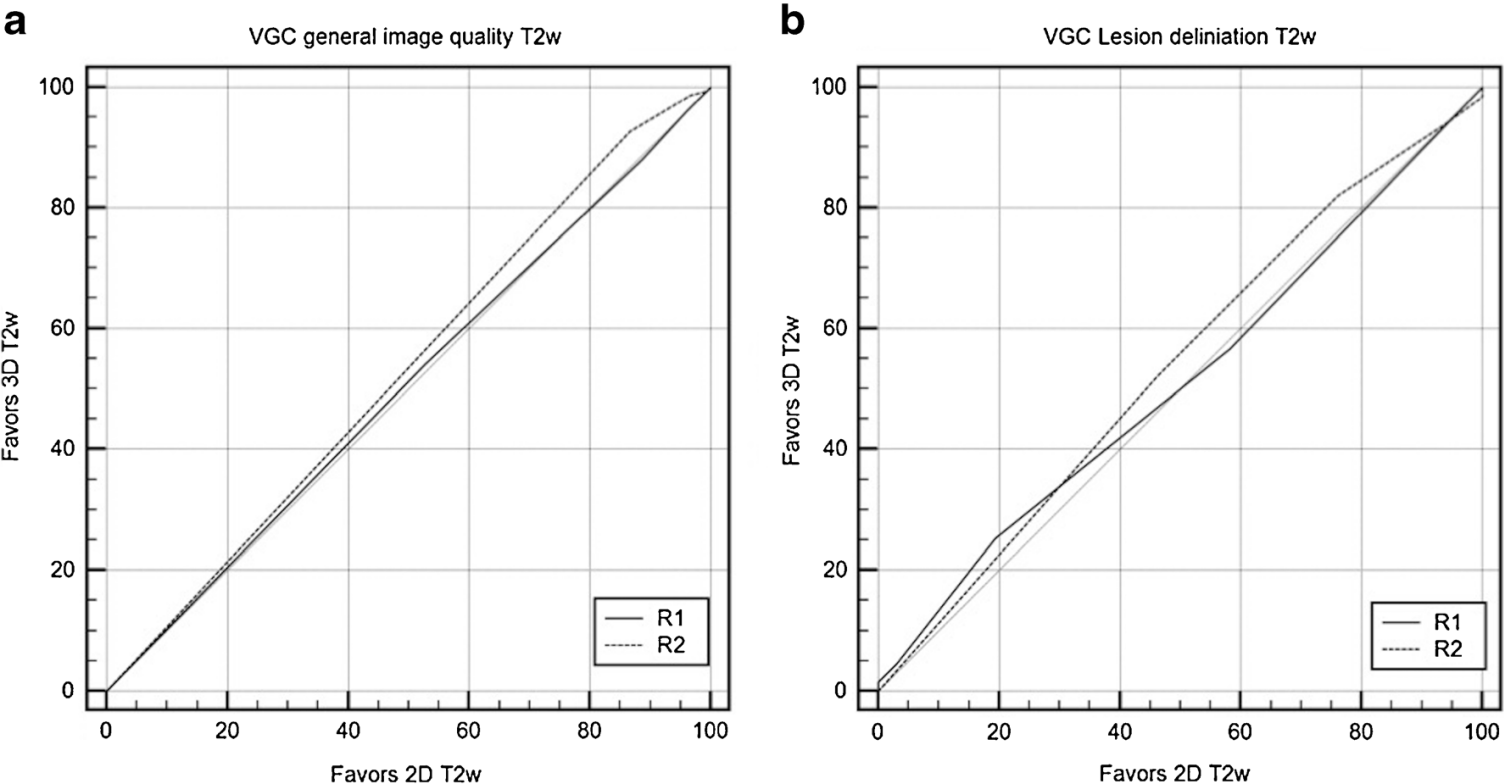
Visual grading characteristics (VGC) curves for image quality assessment and lesion delineation. VGC comparison of 2D and 3D T2w regarding a image quality and b delineation for both R1 and R2. There was no statistically significant difference between the 2D and 3D T2w imaging technique with regard to image quality and lesion delineation (*p* > 0.05). R reader, VGC visual grading characteristics, T2w T2-weighted

Both readers (R1, R2) judged the image quality for 2D T2w (mean score R1, 4.3 ± 0.81; R2, 4.7 ± 0.83) and 3D T2w (mean score R1, 4.3 ± 0.82; R2, 4.7 ± 0.69) as between good and excellent. Overall imaging quality ratings did not differ between 2D and 3D T2w image acquisition (area under the VGC curve R1, 0.504, *p =* 0.894; R2, 0.531, *p =* 0.351). There was no significant difference between both readers, *p =* 0.269 (Fig. 3a).

### Lesion delineation

In our patient cohort, 67 lesions were identified. The lesion delineation was rated as good/excellent by both readers for 2D T2w (mean score R1, 4.16 ± 0.81; R2, 4.19 ± 0.92) and 3D T2w (R1, 4.19 ± 0.94; R2, 4.27 ± 0.94) (Fig. 3b). The area under the VGC curve did not differ between 2D and 3D T2w image acquisition (R1, 0.512, *p =* 0.806; R2, 0.537, *p =* 0.406).

### Lesion characterisation

For lesion characterisation, 53 histopathologically verified patients were further evaluated. Histopathology revealed a prostate carcinoma in 40 patients, and the lesion was benign in 13 patients.

**Fig. 4:**
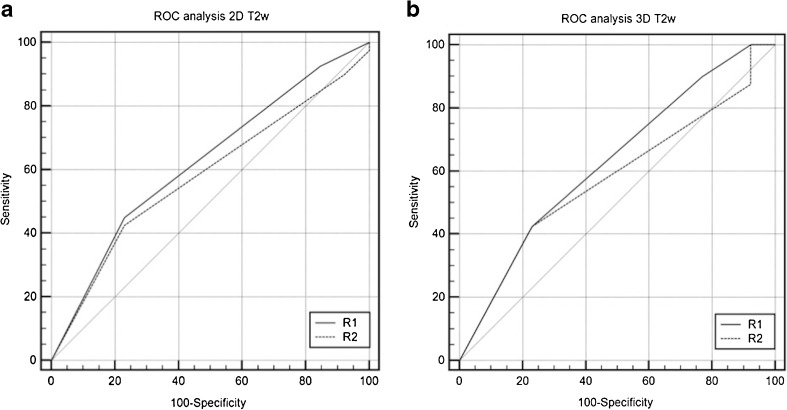
Receiver operating characteristics curves for PI-RADS v2 rating on 2D (a) and 3D (b) sequences. No statistically significant difference was found between both image acquisition methods, *p* > 0.05. R reader, VGC visual grading characteristics, T2w T2-weighted

The diagnostic accuracy for 2D T2-weighted images, as measured by the area under the ROC curve, for both readers was 0.580 (R1) and 0.623 (R2). There was no significant difference (*p =* 0.099) between the readers (Fig. 4a). The area under the ROC curve for 3D T2-weighted images was 0.576 (R1) and 0.629 (R2). There was no significant difference (*P =* 0.1053) between the readers (Fig. 4b).

When comparing the diagnostic accuracy rating for the 2D and 3D T2w images according to the PI-RADS criteria, there was no statistically significant difference between the image acquisition methods: *p =* 0.959 (R1) and *p =* 0.971 (R2).

## Discussion

Our study demonstrated that a faster T2w image acquisition by 3D T2w achieved results as good as standard 2D T2w for overall image quality, lesion delineation and lesion characterisation. There was no statistically significant difference between the two sequences, *p* > 0.05. These results suggest the possibility of using a shorter imaging protocol in patients with suspected prostate cancer, with no decrease in diagnostic accuracy.

Prostate MRI protocols routinely include a 2D T2-weighted sequence in three orthogonal planes while 3D T2w images are still only considered an adjunct to 2D image acquisitions [8]. These sequences are time-consuming and comprise more than a third of our overall protocol. A 3D acquisition would, therefore, be an elegant way to reduce scanning time, as it allows multiplanar reconstructions of the imaging data. The lower in-plane resolution of the 3D T2w sequence (e.g. 0.8 × 0.8 mm in 3D T2w as compared to 0.6 × 0.6 mm in 2D T2w in our study) is compensated by the reduction of partial volume effects that may blur lesion margins as well as lesion and prostate capsules.

Before being applied in the clinical setting, the equivalence of both 2D and 3D acquisitions, in terms of image quality and diagnostic information, needed to be investigated by a head-to-head comparison of both techniques. In our study, we found no difference between the techniques in terms of image quality, lesion delineation and PI-RADS v2 T2w scoring. Our results are supported by a study by Rosenkrantz et al., who showed a substantial agreement between the 2D and 3D sequences for the detection and staging of PCa [12]. The authors performed their scans on a 1.5-T system without an endorectal coil, and subjective image quality scores did differ between both techniques [12]. In contrast to our study, lesions were not scored according to PI-RADS v2 criteria and no VGC analysis was applied. A recent multiple-reader analysis by Westphalen et al. reported a strong individual preference between 2D and 3D acquisitions [13]. The study data was acquired using a 3-T device with an endorectal coil. Quality and lesion delineation were rated on single selected 2D and 3D example images. Although the authors did not look at the entire series, they reported similar delineation ratings with regard to glandular anatomy and cancer lesion depiction between readers and techniques [13].

The 3D technique may also prove beneficial for further evaluation of the neurovascular bundle and for the assessment of extracapsular extension. Panebianco et al. assessed the value of 2D and 3D T2w to assess neurovascular bundle changes after nerve-sparing radical retropubic prostatectomy with regard to erectile function [14]. They concluded that a 3D sequence was better suited in this respect, compared to the 2D approach. In contrast to our study, the investigators used a 1.5-T device with an endorectal coil. Again, the authors showed that the faster 3D approach provided high diagnostic quality. A further 1.5-T (with an endorectal coil) investigation by Cornud et al. found that 3D T2w images provided an accurate depiction of direct and indirect signs of extracapsular extension [15].

In our study, the 3D acquisition required 3 min 45 s, whereas the standard acquisition of three perpendicular 2D T2w planes requires 11 min 14 s. Therefore, the overall protocol in our study could have been shortened by 40% in our scenario (3D T2w + DWI + DCE compared to multiplanar 2D T2w + DWI + DCE, Table 1). According to the estimated scanning time, up to two more patients could be scanned per hour. In addition to cost-effectiveness considerations, reducing the in-bore time per patient is likely to increase patient comfort and, thus, acceptance of the examination. On the other hand, demands on both technical and medical personnel will increase and need to be considered in workflow planning.

**Fig. 5:**
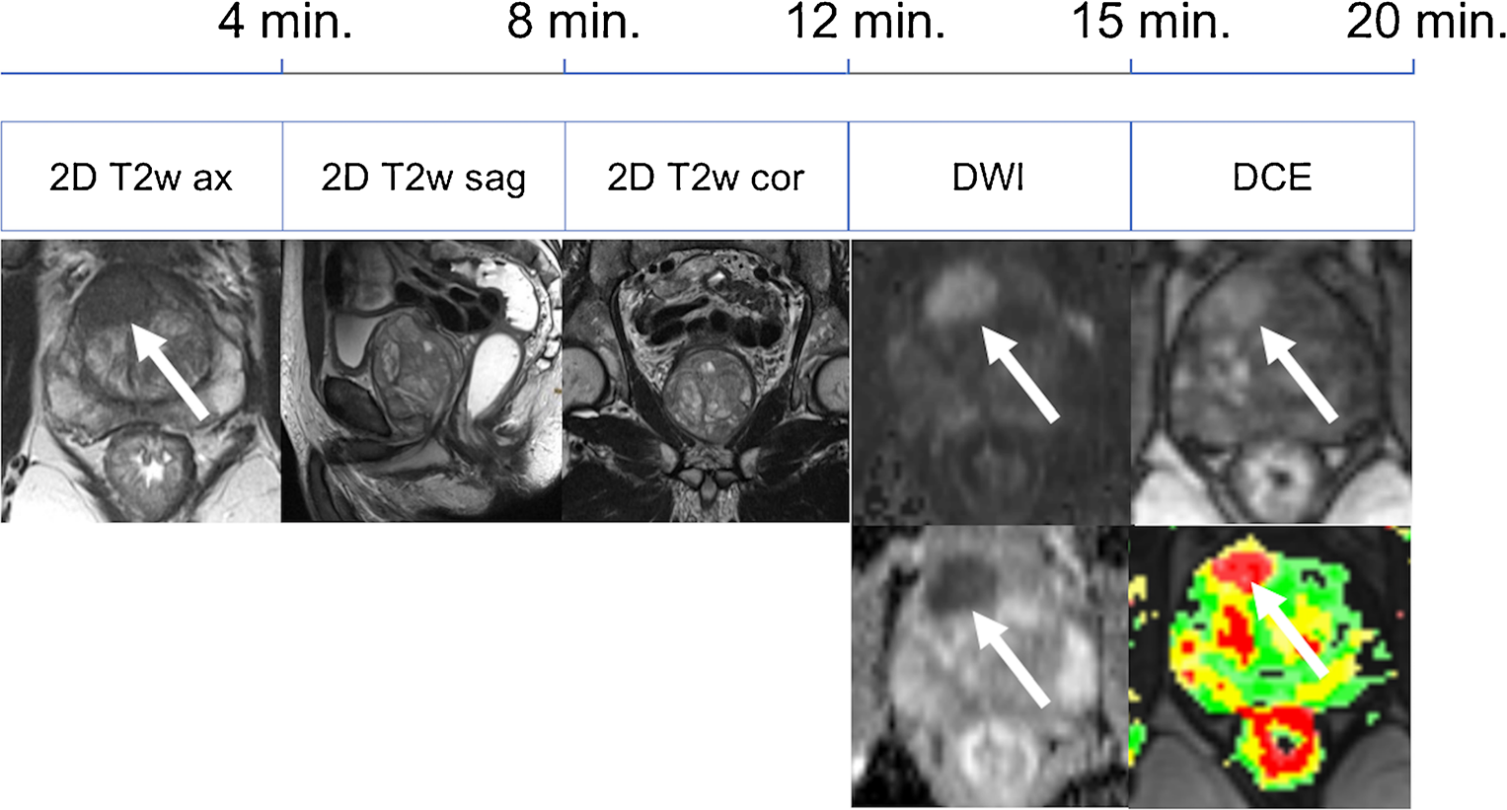
Full multiparametric MRI protocol for detection of prostate cancer

**Fig. 6:**
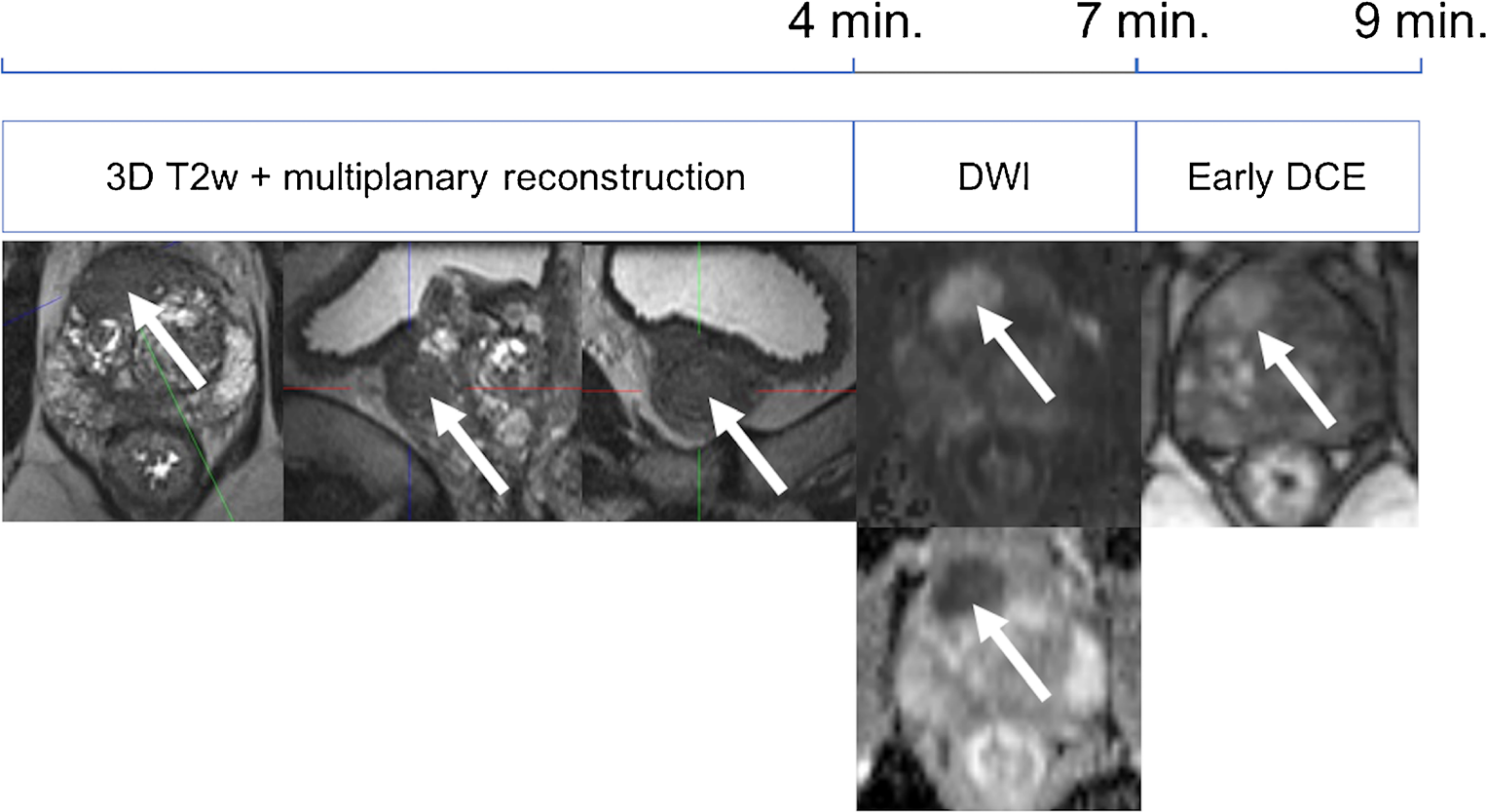
Shortened multiparametric MRI protocol for detection of prostate cancer

There is an ongoing trend toward shortening of multiparametric protocols in the detection and staging of prostate cancer. The initial time-consuming, four-parametric (approximately 50 min) approach has been reduced to a three-parametric standard in the PI-RADS version 2. Several studies had proven that 3D proton MR spectroscopy was of little additional diagnostic value to a three-parameter (T2, DWI and DCE) core protocol [16–18]. By introducing the concept of a dominant sequence in the PI-RADS v2 guidelines—T2w transitional zone and DWI peripheral zone—the role of DCE is a mere additional tool in equivocal peripheral zone lesions. In the peripheral zone, DCE has even been described as having decreased specificity in one study; however, this finding has not been reported by other authors as yet [16, 19–22]. Still, according to the results of Pahwa et al., even minor improvements in PCa detection by using gadolinium contrast media could further increase the cost-effectiveness of prostate MRI [9]. This is because contrast media are relatively cheap when compared to the loss of quality-adjusted life-years if a clinically significant PCa is missed. As PI-RADS v2 guidelines only require early phase DCE images, shortened DCE acquisitions of 1 min 40 s would further reduce scanning time by another 30% without omitting a diagnostic parameter (Table 1; Figs. 5, 6).

One limitation of our study was the limited number of histologically verified lesions in our prospective and consecutive patient cohort. However, the aim of our study was to compare two T2w sequences with regard to overall image quality, quality of lesion delineation and characterisation. As this was a head-to-head comparison of two different T2w acquisition techniques, these were not embedded in different, full MP-MRI readings. Thus, we did not evaluate 2D T2w + DCE/DWI versus 3D T2w + DCE/DWI separately, as the equivalence of both techniques was sufficiently proven by our methodological approach. On the basis of our results, we may thus assume no differences in a three-parametric reading considering either 3D or 2D T2w acquisitions. Of note, the AUCs for both readers were low for lesion characterisation on both T2w sequences. This was expected as histopathology was obtained solely in MRI-detected PI-RADS 4 and 5 lesions causing a patient selection towards suspicious cases and low accuracies. Still, these results further stress the importance of a multiparametric approach to prostate MRI. The protocol time reductions reported here need to be interpreted with care as they may vary to a substantial degree depending on the choice of acquisition parameters. Despite visual grading characteristics being an established and appropriate method for image comparison studies [11, 23, 24], some readers may wish for signal-to-noise and contrast-to-noise ratio comparisons. In the prospective clinical setup, this was not possible because of the use of parallel imaging technology in image acquisition [25].

In conclusion, a single isotropic 3D acquisition of T2-weighted images in multiparametric prostate MRI is equivalent with regard to image quality, lesion delineation and characterisation compared to the longer 2D acquisition in multiple planes. On the basis of these results, multiparametric prostate MRI protocols shorter than 12 min can be designed that allow the examination of up to two patients more per hour in clinical practice.
